# Toward an End-to-End Calibration for Mobile C-Arm in Combination with a Depth Sensor for Surgical Augmented Reality Applications

**DOI:** 10.3390/s20010036

**Published:** 2019-12-19

**Authors:** Sahar Hosseinian, Hossein Arefi, Nassir Navab

**Affiliations:** 1 School of Surveying and Geospatial Engineering, College of Engineering, University of Tehran, Tehran 1439957131, Iran; saharhosseinian@ut.ac.ir; 2 Chair for Computer Aided Medical Procedures & Augmented Reality, Faculty of Computer Science, Technical University of Munich, Boltzmannstr. 3, 85748 Garching b. Munich, Germany; nassir.navab@tum.de

**Keywords:** photogrammetric calibration, interior orientation instability, C-arm devices, 2D X-ray fluoroscopy, image-guided surgery, augmented reality, 3D reconstruction, depth camera.

## Abstract

C-arm X-ray imaging is commonly applied in operating rooms for guiding orthopedic surgeries. Augmented Reality (AR) with C-arm X-ray images during surgery is an efficient way to facilitate procedures for surgeons. However, the accurate calibration process for surgical AR based on C-arm is essential and still challenging due to the limitations of C-arm imaging systems, such as instability of C-arm calibration parameters and the narrow field of view. We extend existing methods using a depth camera and propose a new calibration procedure consisting of calibration of the C-arm imaging system, and 3D/2D calibration of an RGB-D camera and C-arm system with a new method to achieve reliable data and promising accuracy and, at the same time, consistent with standard surgical protocols. For the calibration procedure, we apply bundle adjustment equations with a 3D designed Lego multi-modal phantom, in contrast to the previous methods in which planar calibration phantoms were applied. By using our method, the visualization of the X-ray image upon the 3D data was done, and the achieved mean overlay error was 1.03 mm. The evaluations showed that the proposed calibration procedure provided promising accuracy for AR surgeries and it improved the flexibility and robustness of existing C-arm calibration methods for surgical augmented reality (using C-arm and RGB-D sensor). Moreover, the results showed the efficiency of our method to compensate for the effects of the C-arm movement on calibration parameters. It was shown that the obtained overlay error was improved for the non-zero rotation movement of C-arm by using a virtual detector.

## 1. Introduction

C-arm imaging is a valuable technique to visualize the internal anatomical structure with other instruments and implants in operating rooms to guide surgeries. For orthopedic and trauma surgeries with no navigation technique, many X-ray images are usually needed to be captured. In order to provide guidance for surgeons, one solution is to superimpose desired modalities on the X-ray images during the surgery by using augmented reality (AR). AR provides guidance for the surgeons during the surgery which can decrease the risks of damaging the vital limb and enhance the accuracy of surgery. Besides, this can also reduce the time of surgery and the level of radiation exposure [1].

For C-arm-based surgical AR, calibration is a crucial step as it affects the resulted accuracy of the surgery. This calibration process mainly consists of (a) estimation of the C-arm’s interior orientation parameters which can be performed by using DLT (Direct Linear Transformation) and Tsai’s method [2] as reported in [3,4,5], and (b) AR calibration for the integration of additional sensor such as the depth camera to the C-arm. 

In the following, related works for C-arm surgical AR applications, and the instability of C-arm calibration parameters are explored. Then, the contributions of this study are declared.

### 1.1. Research Overview for AR systems with C-arm

Navab et al. [1] proposed an AR system with video augmentation for C-arm devices in operating rooms by using mirrors. The captured X-ray images were registered with the video images by using a homography matrix. This AR system is limited to 2D guidance. For using 3D information, Habert et al. [6] applied a depth camera augmented on the C-arm by mirror construction, but this construction needed technical modifications to the C-arm device, which contradicted the minimal disruption aim [7]. It also limited the field of view and workspace of the surgeon [1,6]. 

Habert et al. [8] and Habert [7] attached RGB-D sensors to the C-arm X-ray source and applied Zhang’s method [9] with a multi-modal planar phantom in order to obtain the calibration parameters without mirrors. For calibration in the surgical AR navigation with C-arm, current studies, such as [10,11,12,13,14], have also used Zhang’s method [9]. 

In addition, there are some studies, such as [15,16], for registration of Computed Tomography (CT) data to intra-operative 3D surface data in surgical AR. However, these systems are limited to work with only the CT C-arm, which provides 3D volume, and it cannot be applied in operating rooms with 2D Fluoroscopic C-arm imaging systems. 

### 1.2. Instability of C-arm Calibration Parameters

Intrinsic parameters of the C-arm imaging system cannot be considered constant over the image series because of minor torsion of the C-arm caused by the weights of X-ray source and detector [15,17]. Instability in C-arm interior orientation (IO) is an essential factor which severely limits the accuracy of measurements. However, in state of the art methods for most of the researches, the intrinsic camera parameters of C-arm are assumed to be constant. A few investigations, such as [15,17], considered this issue for C-arm calibration. Ignorance of instability in intrinsic parameters can lead to high distortions and errors. 

### 1.3. Contributions

In this paper, we propose a new calibration procedure for C-arm with an attached RGB-D camera for surgical navigation and AR applications. Unlike prior efforts, our method does not rely on DLT or Zhang’s method with a planar phantom for the calibration process in surgical AR. Instead, we apply bundle adjustment with a new 3D multi-modal phantom to improve the robustness of C-arm calibration and transformation parameters between the C-arm system and the RGB-D camera. In addition, we adopt the virtual detector as a reference imaging plane for updating the intrinsic parameters, and we extend it to provide on-line C-arm calibration. 

Our approach provides promising accuracy for the surgical AR application, and it improves the stability of calibration parameters and the flexibility of existing C-arm calibration methods. Moreover, this approach makes it possible to have real-time calibration parameters even if the C-arm is rotated during the operation, in contrast to existing AR methods (of the C-arm and the depth camera). The proposed system has two advantages comparing to the previous versions of AR-based C-arm systems which required that the X-ray source to be positioned above the surgical table: it reduces the radiation exposure to the clinical staff due to scatter [18], and it follows the current clinical practice which increases the chances of acceptance by clinicians. 

In addition, this system can provide a 3D conceptual guidance for surgeons by visualization of the real-time X-ray image on its simultaneous 3D reconstructed model of the scene. In Section 2, the proposed system for C-arm calibration in the surgical AR application will be explained in detail.

## 2. Materials and Methodology 

In this section, the design and implementation of the proposed C-arm calibration procedure are explained. Our calibration approach includes pre- and intra-operative components. The pre-operative step consists of mainly the C-arm intrinsic calibration using a 3D phantom and calculating the transformation between the X-ray and RGB-D camera for the calibration in an AR application during an operation. In the intra-operative component, we use the virtual detector concept [17] to update the intrinsic calibration parameters and an RGB-D camera to continuously recover the pose of C-arm without any marker or external tracking systems. In this system, we prepare calibrated X-ray images during the surgery.

### 2.1. Pre-operative Step

Pre-operative component involves two major steps:

- Setup configuration, which consisted of (1) Designing the 3D phantom for the calibration, (considering geometric network configurations, such as point distribution in object space, convergent imaging, capturing portrait and landscape images, and applying a 3D target field which fills the format of the image to decouple calibration parameters [19]) with a robust procedure considering the limitations of C-arm; (2) Mounting a marker plate for virtual detector concept on the source considering the instability of C-arm calibration parameters; (3) Mounting an RGB-D camera on the C-arm detector for recovering C-arm pose with augmented reality. Then, X-ray images with video and depth images of the new multi-modal phantom could be captured simultaneously, with C-arm in a fixed position.
- Calibration step.

#### 2.1.1. Setup Configuration

To achieve reliable results, we designed a 3D calibration phantom which fills the format of the image, as much as possible, with targets. The imaging network should have a strongly convergent configuration with diversity in camera roll angles. Considering the field of view of the C-arm, size of the image, the desired distance between the X-ray source and the object, we designed a 3D phantom from Lego bricks which are made of ABS (Acrylonitrile Butadiene Styrene) polymer and has the horizontal and vertical pitch of 8 mm and 96 mm. Lego structure has several benefits such as accessibility, low cost, the manufacturing accuracy, and the flexibility to obtain the desired calibration object. 

For creating an appropriate phantom, it is possible to make symmetric desired patterns because of the flexibility in constructions from Legos. We attached stainless steel balls with radii of 0.75 mm and 1.0 mm, as radiopaque markers, on the center of the Lego studs with 8.0 mm and 16.0 mm spaces in each elevation level by drilling the bricks and using glue. For interior orientation, we used points with 8mm spaces, and, at the same time, have a dense test field, filling the format of the X-ray image. 

**Figure 1:**
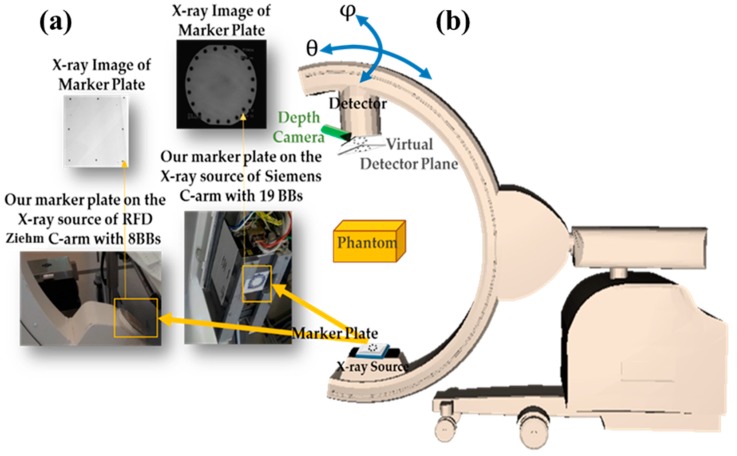
(**a**) The system setup and the proposed marker plates for creating the virtual detector, (**b**) Illustration of C-arm orbital and angular rotations (θ, φ).

Moreover, we should also consider the instability and correlation between calibration parameters during the C-arm movement to improve accuracy. Therefore, we extended the idea of a virtual detector as a reference imaging plane for updating the intrinsic parameters during the surgery because of the instability of parameters [15]. For this purpose, we designed marker plates with several small ball-bearings (BBs), as fiducial markers, attached to the X-ray focus (cf. Figure 1). These markers were projected onto the X-ray image close to the image borders. The proposed marker plate was placed at a fixed position relative to the X-ray source. Therefore, its projection onto the image plane only depends on the intrinsic parameters of the X-ray imaging system. The usage of the virtual detector is explained in Section 2.2.2. The setup of our system and the X-ray images of our proposed marker plates used for creating the virtual detector for C-arm devices are shown in Figure 1. Moreover, C-arm orbital and angular rotations are shown in Figure 1.

#### 2.1.2. C-arm Calibration

X-ray projection geometry could be approximated by the pinhole camera model. C-arm’s interior orientation parameters comprised the X-ray source-detector distance, principal point offsets, aspect ratio, and distortion terms [20,21]. In this research, for determination of calibration parameters of C-arm, nineteen convergent X-ray images in both portrait and landscape orientations were captured and processed while keeping all interior parameters constant during the imaging in the pre-calibration. Moreover, in photogrammetry, having a fixed camera and a portable calibration frame is equivalent to having a fixed calibration frame and a portable camera [22,23]. Therefore, since moving the C-arm could change the intrinsic parameters of C-arm, we moved the calibration phantom to different positions with different orientations, and kept the X-ray source and the detector constant in this step. 

For calculation of C-arm intrinsic parameters, we developed a semi-automatic algorithm in which the user should determine only three points of each level of this 3D phantom to have the same numbering. 

**Figure 2:**
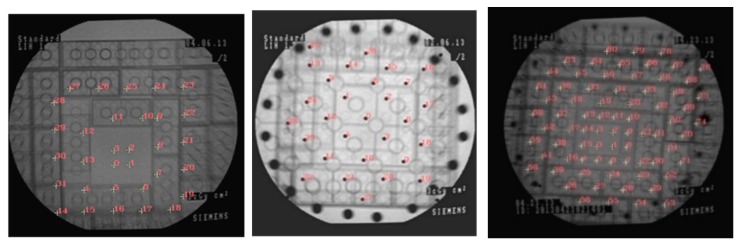
2D point localization in the X-ray images.

For 2D point localization in X-ray images, we extracted the points of the phantom automatically from captured images by using a simple blob detector algorithm (Thresholding, Floodfill, Morphological filtering, and blob detection) using OpenCV library in C++. Figure 2 shows the 2D point localization in the X-ray images of the three applied phantoms. The 3D coordinate of the BBs on the phantom in the object coordinate system was calculated by using the depth camera considering the dimensions of the Lego structure.

The intrinsic parameters of the C-arm X-ray imaging system were calculated using bundle adjustment equations. The mathematical basis of the bundle adjustment is the extended collinearity condition. Collinearity condition is that the 3D object point *(X_i_, Y_i_, Z _i_)*, its homologous 2D X-ray image point *(x_ij_, y_ij_)*, and the perspective center of the image *(XC_j_, YC_j_, ZC_j_)* should lie on a straight line. The perspective projection of a 3D point i on to the X-ray image j can be described as collinearity equations:

(1) $$x_{ij}+ex_{ij}-xp+\Delta x_{ij}=-c\frac{m_{11j}\left(X_{i}-XC_{j}\right)+m_{12j}\left(Y_{i}-YC_{j}\right)+m_{13j}\left(Z_{i}-ZC_{j}\right)}{m_{31j}\left(X_{i}-XC_{j}\right)+m_{32j}\left(Y_{i}-YC_{j}\right)+m_{33j}\left(Z_{i}-ZC_{j}\right)}$$

(2) $$y_{ij}+ey_{ij}-yp+\Delta y_{ij}=c/k\frac{m_{21j}\left(X_{i}-XC_{j}\right)+m_{22j}\left(Y_{i}-YC_{j}\right)+m_{23j}\left(Z_{i}-ZC_{j}\right)}{m_{31j}\left(X_{i}-XC_{j}\right)+m_{32j}\left(Y_{i}-YC_{j}\right)+m_{33j}\left(Z_{i}-ZC_{j}\right)}$$

where c is the distance between the X-ray source and the detector, and *m_j_s* constitute the entries of the rotation matrix of the X-ray image *j*. *k* is the shrinkage factor (changing of the scaling between the axes of the detector), and *ex_ij_* and *ey_ij_* are the random errors. 

In this research, we considered *ex_ij_* and *ey_ij_* as Poisson noise for the X-ray images instead of Normal noise because of the behavior of Quantum noise in Fluoroscopic images [24,25]. In these equations, *xp, yp* were principal point offsets, and Δ*x_ij_,* Δ*y_ij_* were image coordinate perturbation terms or image distortions as interior orientation parameters which considered as follows:

$$\Delta x_{ij}={\mathrm{dx}}_{\mathrm{radial}}+\;{\mathrm{dx}}_{\mathrm{sigmoid}}\;+{\mathrm{dx}}_{\mathrm{affinity}}\;+{\mathrm{dx}}_{\mathrm{local}}$$

$$\Delta y_{ij}={\mathrm{dy}}_{\mathrm{radial}}+\;{\mathrm{dy}}_{\mathrm{sigmoid}}+{\mathrm{dy}}_{\mathrm{affinity}}\;+{\mathrm{dy}}_{\mathrm{local}}$$

where *(dx,dy)_radial_*, *(dx,dy)_sigmoid_*, *(dx,dy)_affinity_*, and *(dx,dy)_local_* were respectively concerned to radial, sigmoid, affinity, and local distortions. We applied similar distortion equations as the ones used in [21] with some modifications as follows: We did not consider decentering distortion since it was ignorable. There were high pincushion and sigmoid distortions for C-arm 2D fluoroscopic imaging. It should be mentioned that the transformation was from the left-handed pixel coordinate system to the right-handed coordinate system. 

We applied bundle adjustment [21,26] for C-arm calibration in the pre-operative step to obtain optimal calibration parameters estimate by using the Levenberg–Marquardt algorithm. For more precise results, initialization was done by applying the depth camera. Using initial estimates of parameters, bundle adjustment refined the pose, the C-arm intrinsic parameters, and the 3D coordinates of the points by minimization of the reprojection error between the observed and predicted image points. The refinement was done by nonlinear minimization. The optimized C-arm intrinsic parameters, relative orientation parameters, and the 3D coordinates of the phantom points were calculated in this step. 

#### 2.1.3. 3D-2D Calibration for Surgical AR

In this step, we used the calculated parameters in Section 2.1.2 for computation of the transformation between the RGB-D camera and the X-ray source for augmented reality in surgery. For this purpose, a procedure to calculate the transformation matrix was implemented by applying the 3D multi-modal phantom. 

In this way, the RGB-D camera should be calibrated at first. The intrinsic parameters of the RGB-D camera could be provided by manufacturers or by calibration methods such as Zhang’s method [9]. Using the depth images, and the intrinsic parameters of the depth camera, the point cloud of each orientation was computed. For noise removal and extracting precise point coordinates, we applied the RANdom SAmple Consensus (RANSAC) Plane Fitting from PCL Library (http://pointclouds.org) for the base plane of the phantom. In this way, the coefficients of the plane were determined. Also, the points of the levels of the 3D phantom were extracted automatically from the RGB images. Using the coefficients of the plane and the extracted target points, the coordinates of the points on the extracted plane were calculated. The equation of the extracted base plane (the plane in the level 0) from the point cloud was: *a X + b Y + c Z + d = 0*, where *a, b, c,* and *d* were the coefficients of the plane, given from RANSAC Plane Fitting algorithm. Since the plane in level j (j > 0) was parallel to the base plane (j = 0) with the height of (*j × H_L_*) from the base plane, the equation of the plane in level j would be:

(3) $$\begin{array}{c} a^{\prime }x+b^{\prime }y+c^{\prime }z\;+d^{\prime }=0\\ a^{\prime }=a,\;b^{\prime }=b,\;c^{\prime }=c,\;d^{\prime }=d-\left(\;j\times H_{L}\right)\times \sqrt{a^{2}+b^{2}+c^{2}}\; \end{array}$$

(4) $$a\;X+b\;Y+c\;Z+\left(d-\left(\;j\times H_{L}\right)\times \sqrt{a^{2}+b^{2}+c^{2}}\;\right)=0$$

where *H_L_* is the height of each level and it was equal to 9.6 mm for all levels, and j was the level number. Then, by considering point cloud coordinate system and the relations between the points and planes of different levels in this phantom, the coordinate of the 3D point i on the Lego phantom in the level j (j > 0) was:

(5) $$\begin{array}{c} Z_{ij}\;=\frac{\;-\;d+\;\left(\;j\;\times \;H_{L}\right)\times \sqrt{a^{2}+b^{2}+c^{2}}}{\left(a\frac{x_{ij}-x_{o}}{f_{x}}+b\frac{y_{ij}-y_{o}}{f_{y}}+c\right)},\;j\in \left\{0,1,2,\ldots ,n_{l}\right\}\\ X_{ij}=\;\left(\left(x_{ij}-x_{o}\right)/f_{x}\right)Z_{ij},\;Y_{ij}=\;\left(\;\left(y_{ij}-y_{o}\right)/f_{y}\right)\;Z_{ij} \end{array}$$

where (*x_0_, y_0_, f_x_, f_y_*) were the RGB-D-camera intrinsic parameters, the principal point offsets, and the focal length in pixel units for x, and y, respectively, calculated by calibration of the depth camera. *(x_ij_, y_ij_)* were the 2D image coordinate of the detected point of *i* (in the level *j*) in RGB image, and *n_l_* was the number of phantom levels {3,4,7}. 

For semi-automatic detection of fiducials in the RGB image, we applied a color-based method (a modified version of CAMSHIFT algorithm) which could be extended by using more robust methods. In this way, the 3D coordinates of targets in the RGB-D camera coordinate system were achieved and corresponded to their 2D coordinates of fiducials in the X-ray images. We created the point cloud of the extracted points by the equation (5), after noise removal with fitting a plane to each level of the phantom with the explained method. With the 2D and 3D correspondences, we used Random Sample Consensus (RANSAC) with the PnP algorithm to estimate the transformation matrix (rotation and translation) between the RGB-D camera and the X-ray C-arm source, by applying the provided interior orientation parameters and simultaneous depth images with X-ray images. Using the coefficients of the plane and the extracted target points, the coordinates of the points on the extracted plane were calculated.

The main results of the pre-operative step were: (a) C-arm calibration parameters; and (b) the transformation between the RGB-D camera and the X-ray source. 

### 2.2. Intra-Operative Step

**Figure 3:**
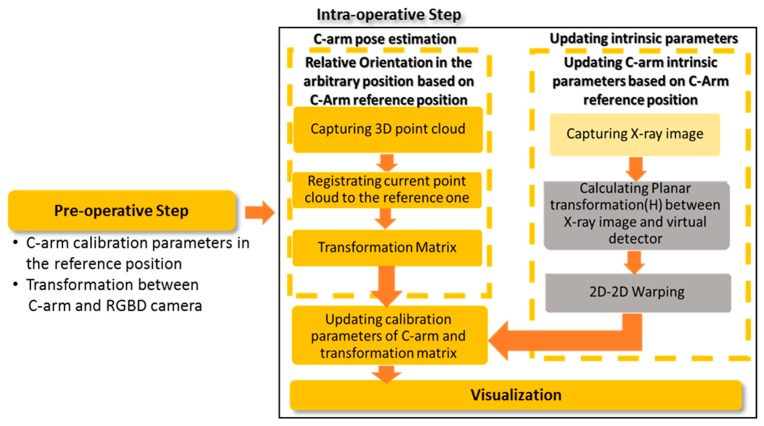
The procedure of intra-operative calibration step.

During the surgery, it is needed to recover the C-arm position and intrinsic parameters before augmentation. In each position, the intrinsic parameters of C-arm should be updated since they are pose-dependent. For this purpose, we considered three components for on-line calibration during the surgery, as shown in Figure 3: (a) C-arm pose estimation, (b) Updating C-arm intrinsic parameters, and (c) Visualization. If C-arm has no movement during the surgery, the pre-operative step is enough, and the augmentation and visualization of X-ray on the 3D model of the surgical scene can be done without updating the calibration parameters.

#### 2.2.1. C-arm Pose Estimation

In this research, considering the limitations of existing external tracking systems evaluated in [27] (such as the arm size for mechanical trackers, and the line of sight problem for optical trackers with their high prices), we applied the point clouds, produced by the mounted depth camera on the detector, for C-arm pose estimation. We updated the C-arm pose with the alignment of the current and the reference point clouds for each C-arm position by using the ICP (Iterative Closest Point) algorithm from the PCL Library (http://pointclouds.org) for C-arm rotation movements of less than 20 degrees because of its simplicity. The ICP is a common rigid registration algorithm, but it requires the initial poses of the two point-sets to be close enough, which is not always possible [28]. Therefore, we applied the Coherent Point Drift (CPD) method by Myronenko and Song [28] for C-arm pose estimation with larger C-arm movements as its efficiency was reported by [28]. In the CPD algorithm, the registration of the two point-clouds was considered as a probability density estimation problem where one point set represented the Gaussian Mixture Model (GMM) centroids, and the other one represented the data points [28]. At the optimum, the point sets would become aligned and the correspondence was obtained using the posterior probabilities of the GMM components [28]. The 3D–3D registration method could be chosen by the user before starting the intra-operative step.

#### 2.2.2. Updating C-arm Intrinsic Parameters during Surgery by Virtual Detector 

The instability of intrinsic parameters of the C-arm during surgery should be considered for intraoperative analysis in each C-arm position. Therefore, the C-arm calibration parameters need to be updated in each C-arm position. 

**Figure 4:**
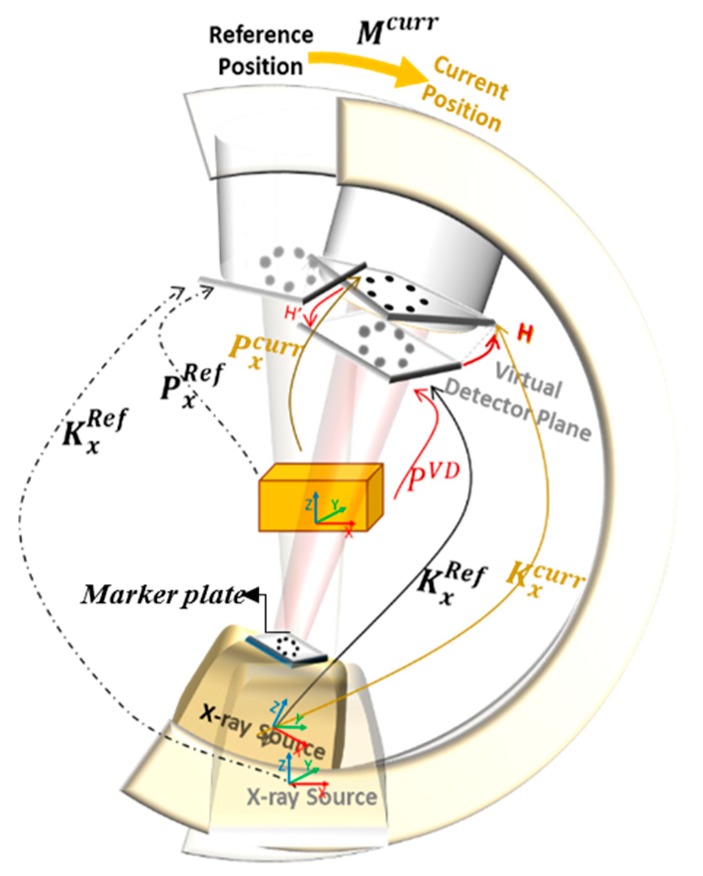
System setup and the transformation chain in the intraoperative step.

Using our approach, X-ray intrinsic parameters would be updated automatically for every C-arm position during the surgery. In this method, we applied a marker plate attached to the X-ray source (as shown in Figure 1, and Figure 4) and computed the planar transformation (**H**) between the X-ray image and the virtual detector. During surgery for the desired position of the C-arm, a new projection matrix was calculated as explained in the following. The C-arm motion $M_{x}^{\mathrm{curr}}$∈ **R**^4x4^ between the current and the reference C-arm position was calculated in the previous step:

(6) $$M_{x}^{\mathrm{curr}}=\left[\begin{array}{cc} R & t\\ 0^{T} & 1 \end{array}\right]$$

If the reference X-ray projection matrix was considered as $P_{x}^{\mathrm{Ref}}\;$∈ **R**^3x4^, then the X-ray projection matrix, $\;P_{x}^{\mathrm{VD}}$ that projects 3D points to the virtual detector plane for the current C-arm position could be calculated by applying the motion **M**_x_^curr^ to the reference X-ray projection:

(7) $$P_{x}^{\mathrm{VD}}=P_{x}^{\mathrm{Ref}}.M_{x}^{\mathrm{curr}}$$

The 2D–2D mapping between the X-ray image and the virtual detector in the C-arm current position (**H** ∈ **R**^3x3^) was computed using the positions of markers on the reference X-ray image and their correspondences on the current detector plane. In the ideal situation with stable intrinsic parameters, this homography matrix **H** should be the identity matrix since the markers were fixed near the X-ray source and their images should be unchanged in different positions. However, due to the instability in C-arm interior orientation parameters and their dependency on the pose, this did not happen for X-ray images, and the homography was not exactly equal to the identity. The changes in positions of these markers in the X-ray images could represent the changes in the intrinsic parameters. Then, the new 3D–2D projection $P_{x}^{\mathrm{curr}}\;$ was achieved by using the 2D–2D planar transformation (**H**):

(8) $$P_{x}^{\mathrm{curr}}=\;H.P_{x}^{\mathrm{VD}}\;$$

(9) $$P_{x}^{\mathrm{curr}}=H.\;P_{x}^{\mathrm{Ref}}.\;M_{x}^{\mathrm{curr}}$$

(10) $$P_{x}^{\mathrm{curr}}=H.\;P_{x}^{\mathrm{Ref}}.\;\left[\begin{array}{cc} R & t\\ 0^{T} & 1 \end{array}\right]\;$$

This could also be written also by updated intrinsic and extrinsic parameters:

(11) $$P_{x}^{\mathrm{curr}}=\;K^{\mathrm{curr}\;}.\;T^{\mathrm{curr}}\;,\;P_{x}^{\mathrm{Ref}}=\;K^{\mathrm{Ref}}.\;T^{\mathrm{Ref}}$$

where $K_{x\;}^{\mathrm{curr}}$ was the real-time updated intrinsic matrix, $T^{\mathrm{curr}}$ is the transformation matrix (extrinsic parameters) for the current position, and $P_{x}^{\mathrm{curr}}\;$ was the updated X-ray projection matrix for the desired position during the surgery. Therefore, we have:

(12) $$P_{x}^{\mathrm{curr}}=H^{\mathrm{curr}\;}.P_{x}^{\mathrm{Ref}}.\;M_{x}^{\mathrm{curr}}=H^{\mathrm{curr}\;}.\;K_{x}^{\mathrm{Ref}}.T_{x}^{\mathrm{Ref}}.\;M_{x}^{\mathrm{curr}}$$

(13) $$P_{x}^{\mathrm{curr}}\;=\;K_{x}^{\mathrm{curr}}.T_{x}^{\mathrm{curr}}$$

Besides, it was possible to calculate a new X-ray projection matrix in the current C-arm position directly by Equation (12). In this way, we could capture calibrated images with known intrinsic and extrinsic parameters during the surgery which was vital, particularly for AR, 3D modeling and measurement applications. In the next section, the experiments done for evaluation of the properties of the applied transformations are expressed. 

#### 2.2.3. Visualization

After updating the calibration parameters, we can visualize the 2D X-ray image on 3D reconstructed data. The transformation (rotation and translation) between the X-ray source and the RGB-D camera was needed for augmented reality and it was calculated as described in Section 2.1.2. If ***a*** denoted the 2D pixel position in the X-ray image, ***A*** represented the corresponding 3D vertex from the phantom, and ***P*** the projection matrix between the X-ray image and the RGB-D camera, we could write:

(14) $$a\;=\;P\;.A=K_{\;}.\left[R\;t\right].A$$

where ***R*** and ***t*** are the rotation and translation matrices between the X-ray source and RGB-D camera coordinate systems, respectively. The intrinsic parameters of the C-arm (***K***) were calculated in the pre-operative step by using bundle adjustment equations. We applied the texture mapping of the X-ray on the 3D reconstruction for visualization using a similar method proposed by [7]. For this purpose, the color of the 3D points in the reconstruction was changed according to the position of their projections into the X-ray image by using the projection matrix. The color of the 3D points in the reconstruction changes to the X-ray color pixel on which a 3D point was projected. In the next section, the experiments done for evaluation of the properties of the applied transformations are expressed. Our proposed system was developed in C++ using OpenCV, OpenGL, and PCL Libraries.

## 3. Experimental Results

We examined our approach on two different C-arm systems as follows: (a) Siremobil Iso-C 3D Siemens C-arm with a field of view of 20° × 20° and X-ray image resolution of 640 × 480 pixels; and (b) RFD3D ZIEHM with the X-ray image resolution of 1024 × 1024 pixels. We mounted Intel RealSense depth camera D435 with the depth image resolution of 1280 × 720 pixels and color image resolution of 640 × 480 pixels near the detector of the C-arm. The achieved results of the implementation of our method for each step are evaluated in the following. 

### 3.1. Experiments and Results of Pre-operative Step

**Figure 5:**
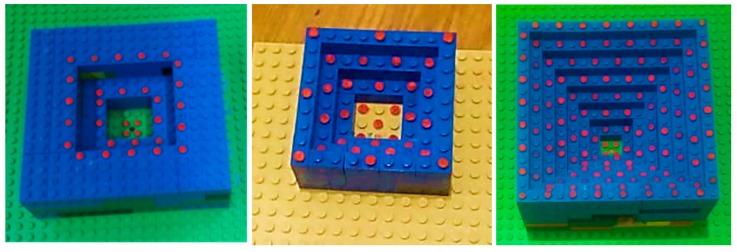
Three-dimensional designed phantoms in this research.

For the pre-operative calibration procedure, we tested the proposed 3D Lego phantoms as shown in Figure 5.

In the first experiment, we made three 3D phantoms with 31, 32, and 95 markers in 3, 4, and 7 levels, respectively, to evaluate the results of interior orientation using phantoms with different numbers of markers, heights, and distributions which their X-ray images are shown in Figure 2. We considered using the minimum numbers of markers to satisfy precision. The space between the markers was 8.0 or 16.0 mm (the space between the studs of Lego bricks). We also applied BBs made of stainless steel with radii of 0.75 and 1 mm for 3D phantoms. In the pre-calibration step, we applied the nineteen X-ray images and computed calibrated parameters with the three phantoms. 

The accuracy of the C-arm calibration in the pre-operative step was evaluated by calculating the root mean square (RMS) of the residuals. The achieved RMSE (Root Mean Square Error) of intrinsic C-arm calibration for RFD Ziehm C-arm was 0.23 pixels with the 3D phantom with seven levels. For accuracy assessment of the calibration method, our proposed method and reference methods were also applied to the same C-arm (Siremobil Siemens C-arm) and the results are shown in Table 1. 

Moreover, the achieved RMSE for 3D–2D calibration between the RGB-D camera and X-ray C-arm using the 3D phantom with seven levels (95 BBs) was 0.58 pixels. This accuracy was promising for surgical augmented reality application and it was comparable to the accuracy of existing methods such as [7] in which the RMSE of 0.79 pixels was obtained for the 3D–2D calibration step.

### 3.2. Experiments and Results of Intra-operative Step

#### 3.2.1. C-arm Pose Estimation 

**Figure 6:**
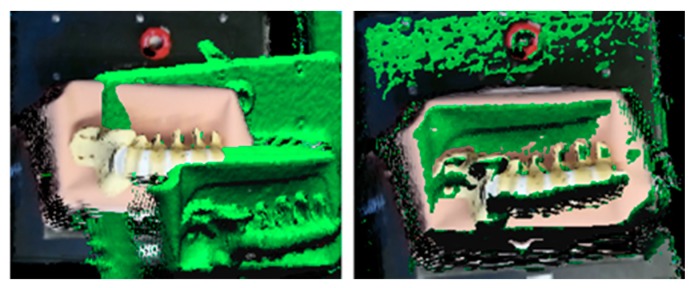
Alignment of the 3D reconstructed models of the scene by using ICP.

After removing noise and filtering the point clouds, we applied the ICP algorithm to align the new point cloud with the reference one for obtaining the relative orientation between the views. In this experiment, the calculated RMS error of the ICP registration algorithm was 28 mm for the C-arm positions with zero angular rotation and orbital rotation of 20 degrees. While the rotation angle between the reference and the desired viewpoint was increasing, the registration error applied by ICP was also increased. Figure 6 shows the alignment of the 3D reconstructed model of the surgical scene from two viewpoints. 

When the angle between viewpoints was larger than 20 degrees, we applied the Coherent Point Drift (CPD) method since CPD led to less registration error than ICP. The calculated RMS error of the CPD registration algorithm was 24 mm for point clouds captured by RealSense D435 in C-arm poses with zero rotation and orbital rotation angle of 35 degrees. 

#### 3.2.2. Results of Updating Intrinsic Parameters and Stability Analysis with a Marker Plate

For making the marker plates, we tested several kinds of materials for the base plate, such as Polyoxymethylene plastic (POM), Ultramid, and Plexiglas. In comparison, Ultramid, and Ultraform (the tradename of POM) had less perturbation and effect on X-rays than Plexiglas. We attached stainless steel BBs with a radius of 0.75 mm on the marker plate with glue as shown in Figure 1. 

**Figure 7:**
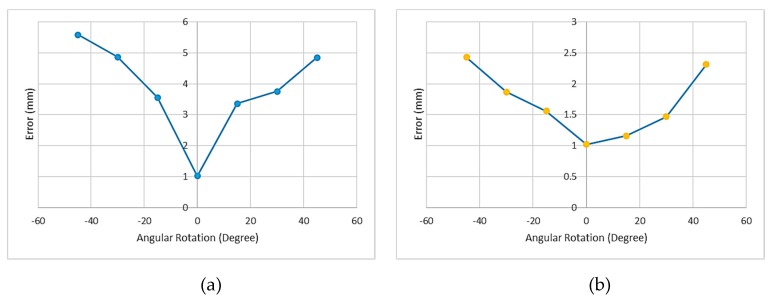
(**a**) The overlay error without using a virtual detector with respect to angular rotation C-arm movement; (**b**) The overlay error with using a virtual detector with respect to angular rotations.

For accuracy assessment of the on-line calibration when applying the virtual detector, we considered the overlay error. For angular rotation angles from −45° to 45°, the mean overlay error for four BBs of the phantom was measured at each orientation. Figure 7 shows the results of the experiment without and with using a virtual detector. Figure 7 indicates that when we used the marker plate for updating the intrinsic parameters, the overlay accuracy was improved for C-arm in different positions. As displayed in Figure 7, the achieved mean overlay error was 1.03 mm for zero rotation movement of the C-arm, while the reference methods such as [7] achieved to the overlay error between 2 and 6 mm. The movements of the C-arm were considered to be only rotations in this research, and the translations were close to zero since it was consistent with the C-arm movements in surgeries.

**Figure 8:**
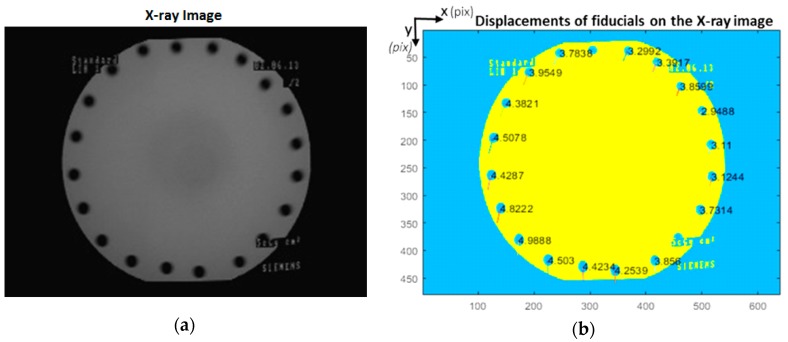
(**a**) The No.6 X-ray image, (**b**) The marker displacements in pixels (multiplied by 5 for visualization of the displacement vectors) of the No.6 X-ray image.

Figure 8b shows the displacements of the fiducials of the marker plane attached to the C-arm source for the No.6 X-ray image (shown in Figure 8a) with the angular rotation of 45 degrees, and orbital rotation of 10 degrees.

For stability analysis of calibration parameters, we applied the Frobenius norm of the computed homography matrix between the reference and the current images for different rotations of the C-arm. The Frobenius norm is the norm of a matrix defined as the square root of the sum of the absolute squares of its elements. The Frobenius norm of the identity matrix is equal to 1.

As mentioned in Section 2.2.2, in ideal conditions in which C-arm calibration parameters were fixed for different C-arm poses, the calculated homography between the reference and the current images would be equal to the identity matrix. In practice, because of the instability of C-arm calibration parameters during the device movements, the calculated homography matrices were not equal to the identity matrix. Therefore, their Frobenius norms would not be equal to 1. 

**Figure 9:**
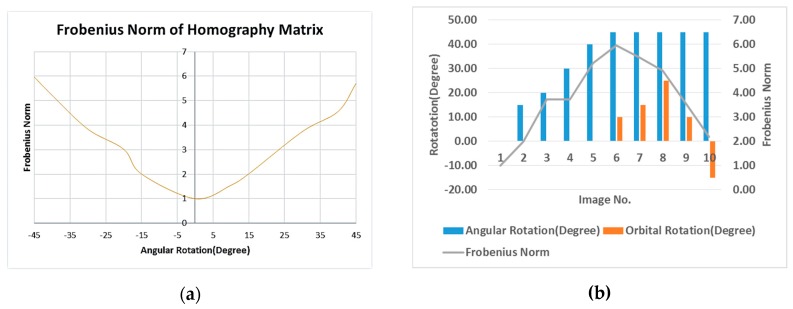
(**a**) Frobenius norm of the homography matrices for X-ray images with respect to only angular rotations, (**b**) Frobenius norm of homography matrix for ten X-ray images with various orbital and angular rotations.

We evaluated the deviation of the Frobenius norm of the calculated homography from 1 for X-ray images depending on the C-arm movements. Figure 9a shows Frobenius norms of the homography matrices for X-ray images with zero orbital rotation and different angular rotations. Figure 9b shows the Frobenius norms of the homography matrices for ten captured images from different positions with various orbital and angular rotations.

**Figure 10:**
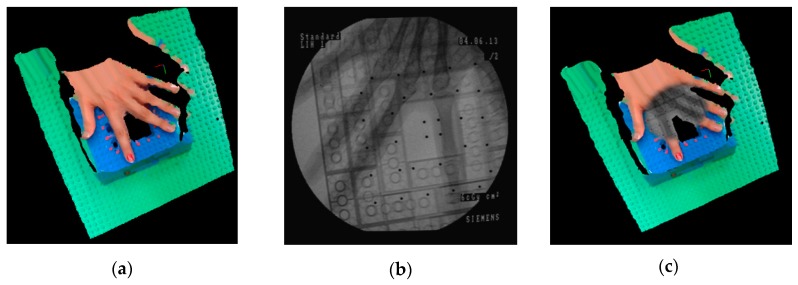
(**a**) 3D reconstructed model of the scene; (**b**) The corresponding X-ray image; (**c**) Visualization of the X-ray image on the 3D reconstruction of the scene by using texture mapping.

Moreover, the simultaneous corresponding X-ray image was visualized on the 3D reconstruction of the scene by using texture mapping with the OpenGL library in C++. Figure 10 shows the result of the visualization and augmentation in this research.

For the implementation of our system, the setup of Intel (R) Core i7 2.80GHz processor with NVIDIA Geforce GTX 1050 Ti was applied. The achieved time performance for the intra-operative step with visualization was 148 ms which was promising for AR surgeries, and it could be considered in real-time.

## 4. Discussion

We evaluated the results of the proposed system for C-arm calibration in surgical augmented reality applications. For the pre-operative step, the C-arm calibration results indicated that the number and distribution of the markers of the 3D phantoms in all axes were important issues. When our C-arm calibration method was applied by the proposed 3D phantom with 95 markers, it led to comparable accuracy (with RMSE of 0.33 pixels) to the reference calibration approaches such as [7] (with RMSE of 0.37 pixels) for surgical AR on the same C-arm device (Siremobil Siemens). The resulted RMSE of C-arm calibration was 0.23 pixels by using our method (with seven-level phantom) for Ziehm C-arm which had lower distortion than Siremobil Siemens. The achieved RMSE of 3D-2D calibration between the RGB-D camera and Siemens C-arm by using the 3D phantom with seven levels was comparable to the existing methods such as [7] on the same C-arm. Moreover, the number of markers of the proposed calibration phantom was 95 markers, while the applied 2D phantoms in reference methods had more than 200 markers. In this way, if we increase the number of the phantom markers, with considering the distribution in three axes and filling the format of the X-ray images, it is expected that the calibration accuracy would be improved more. However, the distance between the depth sensor and the surgical scene in this experiment was small and this decreased the quality of 3D reconstruction of the scene in comparison with using RealSense or Kinect 2 in distance of 40 cm or more as applied in the reference methods such as [7,8]. For 2D target localization in the pre-operative step, all markers with a radius of 1 mm were automatically detected, while a few markers with a radius of 0.75 mm could not be detected and the user intervention was needed. Therefore, the size of markers could affect the result of automatic target localization.

In the following, the intra-operative calibration procedure is discussed. For recovering the C-arm pose during the surgery, we tested ICP and CPD registration algorithms. When the rotation angle between the viewpoints was large or the calculated RMSE of registration was more than 30 mm, we applied the Coherent Point Drift (CPD) method for registration. CPD algorithm was more efficient than ICP in the experiments. However, we considered that using 3D-3D registration of point clouds for recovering the C-arm pose during the surgery is difficult since the surgical scene is changing during the operation. This C-arm pose estimation method can be sufficient for training surgeries. In addition, the new generation of C-arm devices can report the C-arm pose to the user automatically, and they can be applied directly in the proposed calibration procedure. 

For updating C-arm intrinsic parameters during the surgery, we made marker plates with considering several aspects such as (a) the material of the plate and (b) the size, (c) the material, (d) the number, and (e) the distribution of the fiducials to stabilize the interior orientation parameters. For stability analysis and evaluation of on-line calibration, we considered the Frobenius norm of the matrices and the overlay error. This evaluation showed that updating the intrinsic parameters of C-arm systems (by using the extended virtual detector method) would improve the overlay accuracy during surgery. 

## 5. Conclusions

In this paper, we introduced a solution for the calibration of an integrated C-arm with an RGB-D sensor for surgical AR. Our calibration solution was developed in C++ and it consisted of two components: pre-operative, and intra-operative calibration. In the pre-operative step, the calibration parameters of the C-arm imaging system were mainly computed by using bundle adjustment with a 3D Lego phantom. In this step, the transformation between the C-arm and the RGB-D camera was also calculated. By evaluating the pre-operative calibration procedure for several phantoms, the highest accuracy was obtained for the 3D phantom with more fiducial markers and height levels (95 markers with seven height levels in this experiment). The assessment of the calibration phantoms also indicated that the number and distribution of the fiducials of the phantoms were major issues for C-arm calibration in surgical AR.

For the intra-operative step, we extended a virtual detector for updating calibration parameters during the surgery due to the mechanical sagging of the C-arm gantry (caused by the rotations). Evaluation of the proposed system was done in Experimental Result and Discussion Sections. The results proved the feasibility of the proposed method for on-line C-arm calibration. 

We have shown that by applying the proposed method using bundle adjustment equations and on-line calibration, the real-time 3D reconstruction of the surgical scene can be registered to the corresponding X-ray image with comparable accuracy even by using fewer markers in the calibration phantom and it resulted in accurate real-time AR overlay. This would provide guidance for surgeons, and it also could prevent high radiation doses for capturing many images in order to conduct the surgery. 

For future work, it would be recommended to apply bundle adjustment by using a 3D phantom with more fiducials, considering the distribution of the markers to achieve more accurate results. Moreover, calibration parameters of C-arm and the transformation between sensors, as the results of this solution, are also needed for different medical applications, such as surgical navigation, surgeon hand tracking, relevance-based overlay, surgical tool tracking, and 3D reconstruction of the surgical subjects. 

## Figures and Tables

**Table 1:** Results of error analysis of our C-arm calibration method using different 3D phantoms in comparison to current reference methods.

Proposed Method with designed phantoms:	**C-arm** **device**	**Phantom** **type**	**Material**	**Number of levels**	**Number of BBs**	**RMSE** **(Pixel)**
	Siemens Siemens Siemens Ziehm	3D 3D 3D 3D	Lego Lego Lego Lego	3 4 7 7	31 32 95 95	1.21 0.96 0.33 0.23
Previous Reference Systems: [7] CAMC [12] Zhang’s method [12]	**C-arm** **Device** Siemens Siemens Siemens	**Phantom** **type** 2D - 2D	**Material** PCB (Printed Circuit Board) - PCB (Printed Circuit Board)	**Number** **of levels** 1 1	**Number** **of BBs** >200 >200	**RMSE** **(Pixel)** 0.37 1.02 0.48
